# Evaluation of the Alterability of Natural and Artificial Agglomerated Stones Produced in Epoxy Matrix

**DOI:** 10.3390/polym18101164

**Published:** 2026-05-09

**Authors:** Evanizis Dias Frizzera Castilho, Mônica Castoldi Borlini Gadioli, Mariane Costalonga de Aguiar, Markssuel Teixeira Marvila, Carlos Mauricio Fontes Vieira, Sergio Neves Monteiro, Afonso Rangel Garcez de Azevedo

**Affiliations:** 1 Federal Institute of Espírito Santo—IFES, Campus Cachoeiro de Itapemirim, Av. Engenheiro Fabiano Vivacqua, 1568, Cachoeiro de Itapemirim 29311-970, ES, Brazil; evanizis@ifes.edu.br; 2 Ministry of Development, Industry, Commerce and Services—MDIC, Esplanada dos Ministérios, Brasília 70053-900, DF, Brazil; mborlini@cetem.gov.br; 3 Centre for Mineral Technology—CETEM, Cachoeiro de Itapemirim 29311-970, ES, Brazil; maguiar@cetem.gov.br; 4 Rio Paranaíba Campus, Federal University of Viçosa, Rodovia BR 230 KM 7, Rio Paranaiba 38810-000, MG, Brazil; markssuel.marvila@ufv.br; 5 Advanced Materials Laboratory and LAMAV, State University of the Northern Rio de Janeiro—UENF, Av. Alberto Lamego, 2000, Campos Dos Goytacazes 28013-602, RJ, Brazil; vieira@uenf.br; 6 Department of Materials Science, IME—Military Institute of Engineering, Square General Tibúrcio, 80, Rio de Janeiro 22290-270, RJ, Brazil

**Keywords:** artificial agglomerated stones, ornamental stone waste, epoxy resin, alterability

## Abstract

The growing demand for sustainable solutions in civil construction has driven the use of industrial waste in the formulation of new materials. This study evaluated the alterability of artificial stone slabs produced with 87% ornamental stone waste (Dumont Quartzite and Preto São Gabriel Granite) and 13% epoxy matrix, using the vacuum vibro-thermo-compression technique. Alterability was tested against staining agents, chemical attack, wetting and drying cycles, UV radiation, salt crystallization, three-point flexural strength test after freeze–thaw cycles, and natural weathering. Quantitative results revealed high physical stability, with mass loss varying only between 0.11% and 0.23% in wetting and drying cycles. In salt crystallization, mass loss ranged from 0.38% to 0.47%, lower than the rates of 0.87% and 1.36% reported in the literature for similar materials. Regarding mechanical performance, freeze–thaw cycles caused reductions in flexural strength between 11.66% and 31.59%; however, the stones maintained final strength values above 20 MPa, classifying them as very high-strength materials. The results indicated good physical and chemical stability of the materials, with low mass loss and preservation of mechanical properties, except under UV radiation and natural weathering, which caused significant chromatic alterations. The data obtained demonstrate the viability of applying these stones in indoor environments, promoting the valorization of waste and contributing to the circular economy.

## 1. Introduction

The ornamental stone industry plays a significant role in the Brazilian economy, particularly in the state of Espírito Santo, which accounts for approximately 82% of the country’s exports in this sector and concentrates nearly all primary processing activities, such as slab sawing. According to the Brazilian Ornamental Stone Association, Brazilian exports of ornamental and coating natural stone materials reached USD 1.26 billion and 2.05 million tons in 2024 [1,2].

Due to the large production of ornamental stones in the country, waste generation is very high. Given this scenario, the use of ornamental stone waste in the manufacture of agglomerated stones emerges as a promising alternative to mitigate environmental impacts and add value to the industry’s byproducts.

Agglomerated stones can be classified as particulate composites, where fine ornamental stone waste serves as reinforcement, and the matrix is composed of polymeric binders (e.g., epoxy or polyester resins) or cementitious materials. The production of agglomerated stones from waste materials represents a practical and innovative application of composite technology, incorporating mineral particles with relevant physical and chemical characteristics into a matrix that provides cohesion, moldability, and resistance [3,4].

The research focused on evaluating their technical performance and long-term alterability. Specifically, for artificial agglomerated stones, alterability tests have been employed to assess the physical, aesthetic, and structural stability of the materials over time [4]. These analyses are essential to ensure that products developed with industrial waste not only perform at levels comparable to conventional materials but also comply with technical standards and market expectations.

Although studies have shown that artificial agglomerated stones can exhibit physical and mechanical properties comparable to those of natural stones [5,6,7], research focused on the long-term alterability of these materials under real usage conditions remains limited.

The production of artificial stones from industrial waste demands a thorough understanding of the properties of the raw materials involved. In this context, alterability tests that simulate natural degradation processes over time are essential for assessing the alterability and stability of these composites. Such tests enable the identification of structural changes, mass loss, chromatic alterations, and other signs of deterioration, thereby providing critical insights for the optimization of formulations and for ensuring the safe application of these materials in architectural and industrial settings.

Therefore, the use of ornamental stone waste in the production of innovative composite materials, coupled with evaluations of their resistance to physical and chemical degradation, emerges as a promising strategy to align environmental sustainability with technical viability and technological innovation in the construction materials sector.

This study aims to evaluate the alterability of artificial agglomerated stones produced with ornamental stone waste, with a focus on understanding their behavior over time. The goal is to contribute to the development of more sustainable materials that exhibit improved technical performance.

This study adopts a comprehensive and comparative approach involving both natural stones and artificial agglomerated stones with an epoxy matrix, subjected to various alterability tests: ultraviolet (UV) radiation, salt crystallization, and freeze–thaw cycles. The originality of the study lies not only in the diversity and combination of these tests but also in the application of sustainable agglomerates formulated with an epoxy matrix, for which there is a lack of systematic data regarding performance and long-term alterability.

The results obtained contribute to filling a gap in the technical-scientific knowledge base, offering unprecedented support for the development of standards and guidelines for the safe and efficient application of these materials, especially in indoor environments. In this way, the present work advances the understanding of the alterability of sustainable agglomerated stones, fostering innovation and sustainability in the civil construction sector.

Despite advancements in the development of artificial agglomerated stones produced from ornamental stone waste and polymeric matrices, research still focuses primarily on the initial physical and mechanical characterization of these materials, emphasizing properties such as flexural strength, water absorption, and density. In this context, studies that comprehensively evaluate the weatherability behavior of these composites remain limited, especially when subjected to different degradation agents and in direct comparison with natural stones. Aspects related to long-term alterability, particularly under combined exposure conditions such as UV radiation, salt crystallization, wetting and drying cycles, and natural weathering, are still poorly explored in the literature.

The long-term performance of artificial agglomerated stones, particularly those based on epoxy resins, has become an important research topic due to their increasing application in civil construction. Although several studies have demonstrated that these materials can achieve satisfactory physical and mechanical properties, their alterability under environmental exposure remains a critical aspect that is not yet fully understood. In particular, the alterability of epoxy-based artificial stones can be influenced by factors such as ultraviolet (UV) radiation, humidity variations, chemical agents, and salt crystallization, which can progressively affect both their aesthetic and structural properties.

Previous studies have investigated the alterability of artificial agglomerated stones under specific degradation conditions [4,7,8]. For example, some authors have evaluated resistance to chemical attack and pigmentation, highlighting the influence of the polymer matrix on surface stability and resistance to aggressive agents [4,7]. Other studies have focused on salt crystallization and wetting and drying cycles, demonstrating that mass loss and microstructural degradation are frequently associated with porosity and matrix–particle interface quality [7,8]. In addition, studies involving exposure to UV radiation have reported chromatic changes and surface degradation, mainly attributed to photo-oxidation of epoxy resins [9].

Although these studies provide relevant contributions to understanding the behavior of artificial stones, the results are still fragmented in relation to the different degradation agents evaluated. In many cases, the performance of epoxy-based artificial agglomerated stones is analyzed under specific conditions, which hinders a broader understanding of how different degradation processes can act simultaneously on the material. Furthermore, direct comparisons between artificial and natural stones, conducted under equivalent experimental conditions, are still relatively limited, which restricts a more consistent assessment of the relative performance between these materials.

In this context, it becomes necessary to expand investigations into the alterability of these materials, considering different degradation mechanisms within the same experimental approach. The joint evaluation of the influence of physical, chemical, and environmental factors can provide more consistent information on performance over time, contributing to the development of artificial agglomerated stones with greater alterability and reliability for application in civil construction.

## 2. Materials and Methods

### 2.1. Materials

Two different types of waste generated during the sawing of blocks into slabs using diamond multiwire gangsaw equipment were used in this research. The “Dumont” quartzite and “Preto São Gabriel” granite waste were donated by companies located in (Cachoeiro de Itapemirim, ES), Brazil.

The solid waste from large stone pieces produced during block sawing (external parts of the blocks) was hammered and subjected to a jaw crusher to obtain coarse particles and ground in a porcelain disc mill to obtain medium particles.

The fine waste was collected in liquid form, called fines from the processing of ornamental stones (FIBRO). This waste was dried at room temperature around 25 °C to remove excess moisture and then in an oven for 24 h at a temperature of 110 °C and then subjected to the Gral and Pistilo for better separation of the particles.

Subsequently, the waste was sieved in a benchtop vibrating sieve (Table 1). To perform the sieving, 10, 40 and 200 mesh sieves were used, according to the technical standard NBR 7181 [10].

The resin used was Epoxy 2001, liquid in appearance, resulting from the reaction of Epichlorohydrin with Bisphenol-A, viscosity 1.16 g/cm^3^. The hardener 3154, based on polyamine, has a viscosity of 1.005 g/cm^3^. The mixing ratio (resin:hardener) is 100:50 by weight. Both components were supplied by Redelease manufactured in (São Paulo, SP), Brazil.

#### Determination of the Particle Size Composition of the Highest Packing

**Figure 1:**
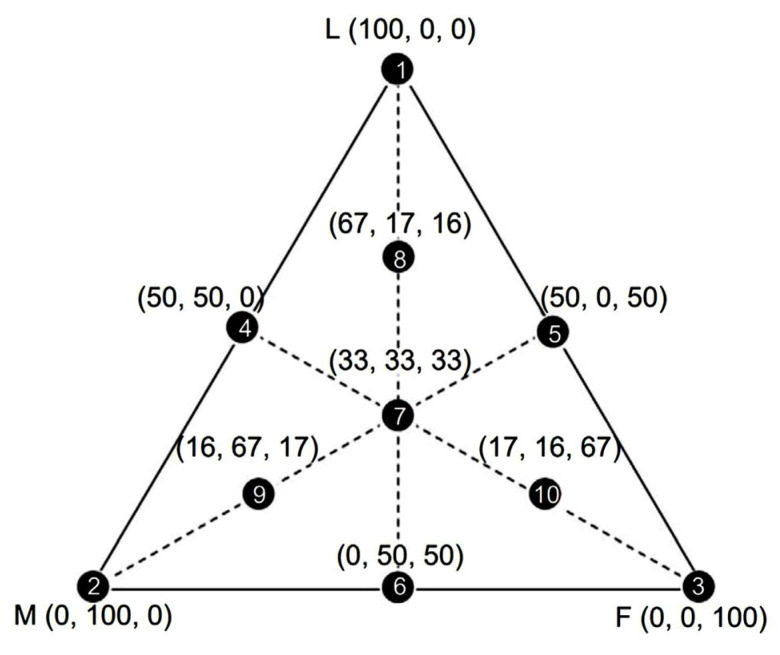
Geometric illustration of the ternary diagram of the simplex model. Each point corresponds to a composition of coarse (L), medium (M) and fine (F) particles. The numbers in parentheses indicate the weight percentages of coarse, medium and fine particles, respectively [8].

To determine the granulometric composition with improved packing, three granulometric bands (coarse, medium and fine) of the ornamental stone waste were used. Based on these three granulometric bands, ten different mixtures were developed, applying the “simplex-centroid model”. The ternary diagram, represented geometrically in Figure 1, highlights each point as a mixture with a specific composition.

The test was based on NBR 16843 [11], aiming to determine the greatest proportion of packaging of the waste that was used, that is, to determine which of these mixtures presented the maximum dry apparent density.

The mixtures of different proportions were placed in a packaging container, where a 10 kg weight was attached to the top. This container was then positioned on the Benchtop Vibrating Sieve equipment. The mixtures were vibrated for 10 min at a frequency of 60 Hz. This procedure was repeated three times for each of the 10 compositions of the simplex model. After vibration, the mixture was weighed and the vibrated density calculated. The highest dry apparent density is related to the greatest particle packaging, that is, the highest vibrated density of the mixtures.

### 2.2. Methods

**Figure 2:**
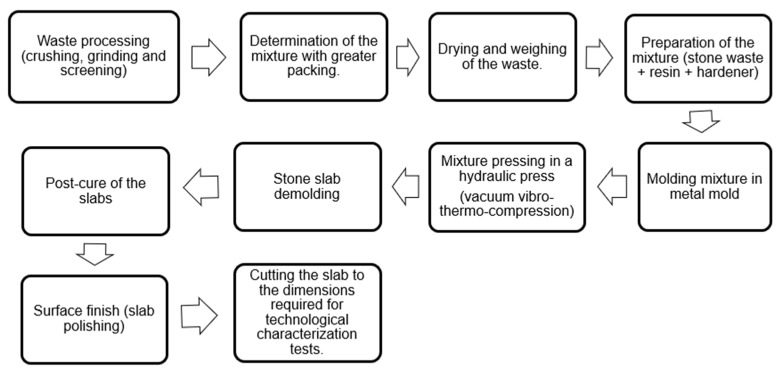
Stages of the manufacturing process for artificial agglomerated stone slabs.

The manufacturing process of the artificial agglomerated stone slabs was carried out according to the steps illustrated in Figure 2.

#### 2.2.1. Waste Preparation and Production of Agglomerated Stone

The natural stone waste with the highest apparent dry density was dried in an oven at a temperature of 110 °C for 24 h to eliminate moisture. To manufacture the artificial stone slabs, a composition containing 87 wt% by mass of Preto São Gabriel granite waste and Dumont Quartzite and 13 wt% by mass of a mixture of epoxy resin and hardener was used. After drying, the waste was manually mixed with the resin and catalyst and homogenized for a period of 2 min to ensure uniform distribution of the components.

**Figure 3:**
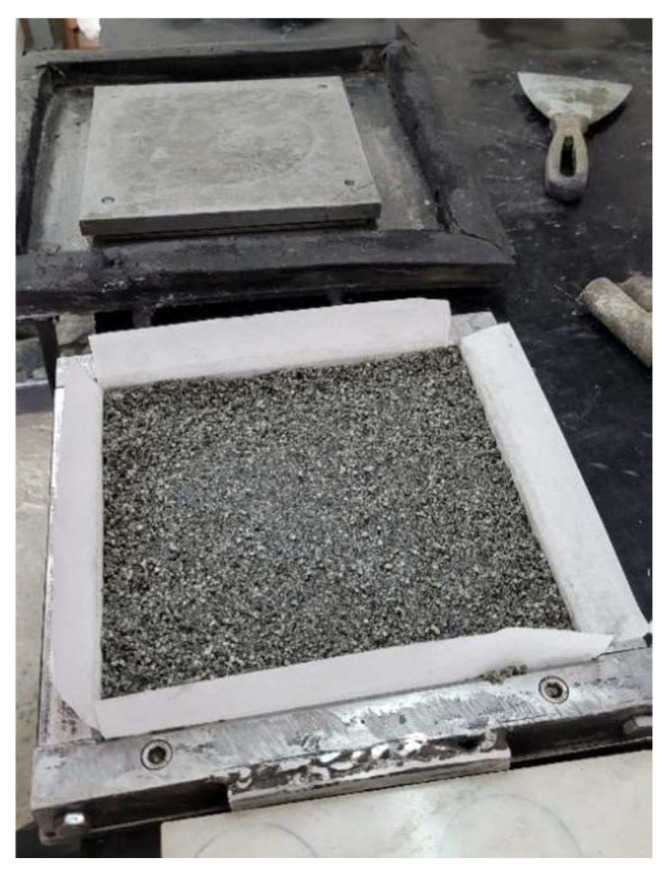
Metal mold.

**Figure 4:**
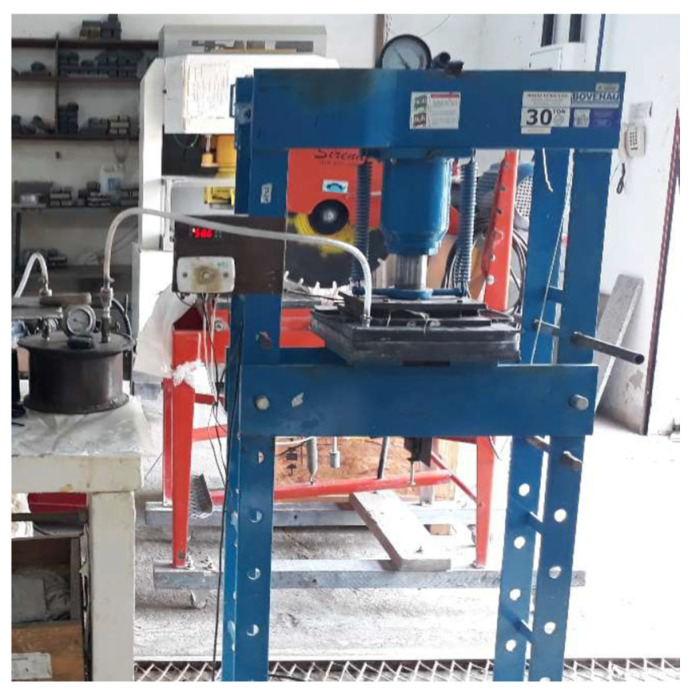
Hydraulic press.

The formed mass was then transferred to a metal mold measuring 200 mm × 200 mm × 10 mm (Figure 3) and pressed in a hydraulic press (Figure 4) under a compaction pressure of 3.68 MPa. At the same time, the system was heated to 90 °C for 20 min, coupled to a 600 mmHg vacuum system and subjected to vibration at a frequency of 60 Hz. After these processes, the plates obtained were cured at room temperature for a period of seven days. They then underwent the polishing and cutting stages to the dimensions specified by the technical standards for technological characterization tests.

#### 2.2.2. Technological Characterization of Stones

To perform the technological characterization of the stones, four types of samples were used: (i) natural stone of Dumont Quartzite (NSQD); (ii) natural stone of Preto São Gabriel Granite (NSPSG); (iii) artificial agglomerated stones of Dumont Quartzite (ASQD); and (iv) artificial agglomerated stones of Preto São Gabriel Granite (ASPSG).

**Figure 5:**
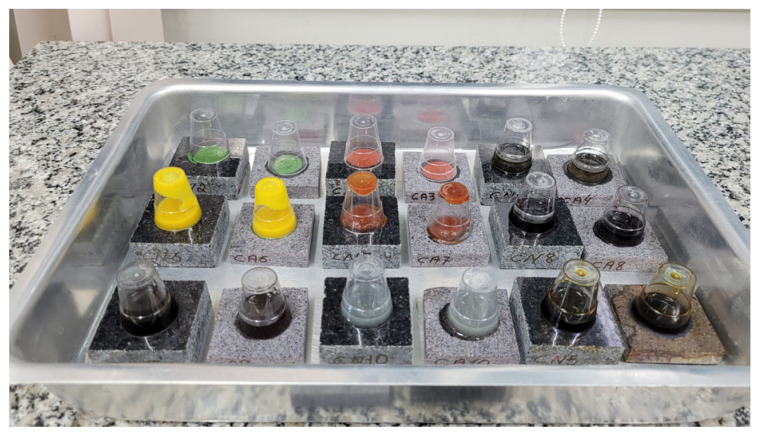
Test specimens subjected to staining.

To determine stain resistance, 10 test specimens with dimensions of 50 × 50 × 10 mm were used. This test was performed in accordance with NBR 10545-14 [12] in an adapted manner. The samples were exposed for 24 h to penetrating agents (Cr_2_O_3_—green and Fe_2_O_3_—red), an oxidizing agent (iodine), a film-forming agent (olive oil) and household products such as wine, coffee, ketchup, mustard and lemon juice (Figure 5). The material was classified according to how easy it was to remove stains after the cleaning steps described in the standard.

**Figure 6:**
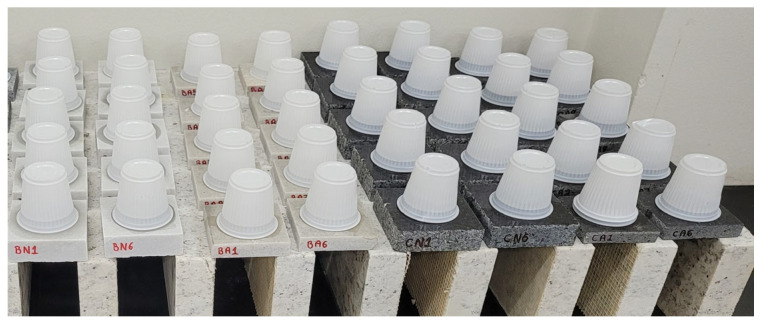
Chemical attack test.

To determine resistance to chemical attack, ten stone samples, each measuring 50 × 10 mm, were used and exposed to various chemical agents (Figure 6). The surfaces of the samples were carefully inspected before and after the tests to detect possible color changes, dissolution or mineral detachment. The chemical reagents used were ammonium chloride (NH_4_Cl), sodium hypochlorite (NaClO), citric acid (C_6_H_8_O_7_), lactic acid (C_3_H_6_O_3_) and acetic acid (C_2_H_4_O_2_), with exposure for 24 h. In addition, they were exposed to hydrochloric acid (HCl), potassium hydroxide (KOH) and deionized water for 96 h, following the guidelines of NBR 16596 [13] for stone coating. The exposure time to the reagents varied from 24 to 96 h, as shown in Table 2.

**Figure 7:**
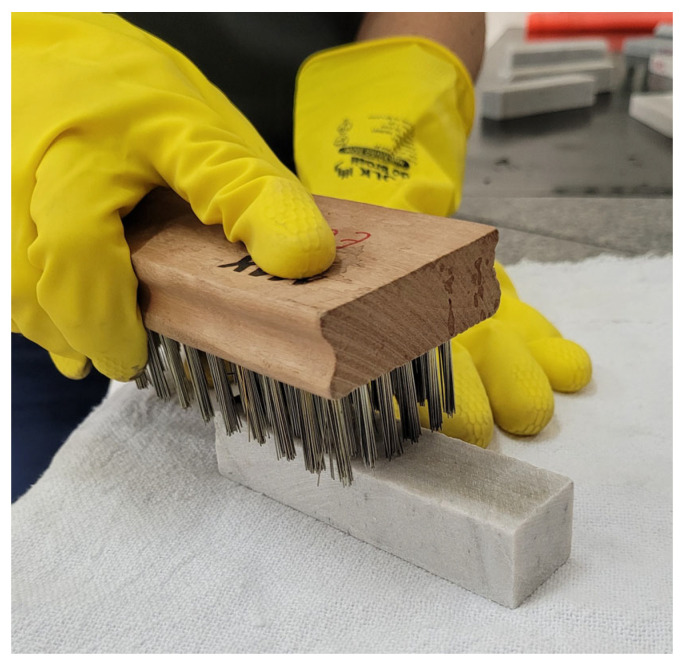
Test specimens being brushed.

To evaluate alterability by wetting and drying, five test specimens measuring 100 × 25 × 10 mm were used and subjected to ten wetting and drying cycles, according to the procedure described in NBR 13554 [14]. In each cycle, the test specimens were brushed with a steel strip brush (Figure 7).

**Figure 8:**
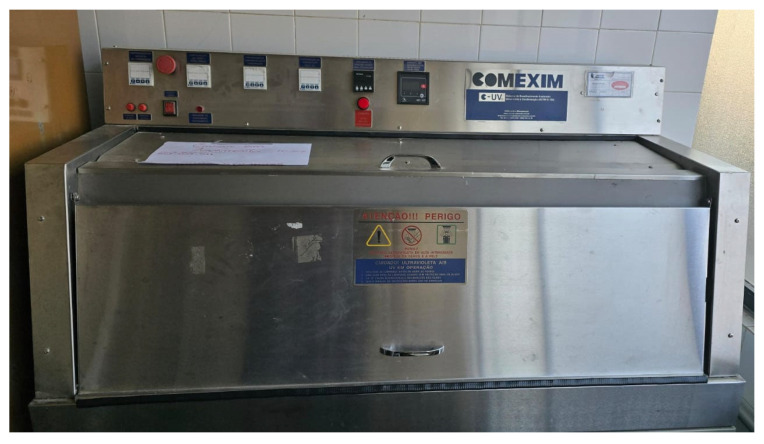
UV-ray camera.

To evaluate the resistance to UV radiation and water condensation by means of accelerated testing, a COMEXIM brand (São Paulo, SP, Brazil UV test chamber model ASTM G154 was used, equipped with a sample holder and eight 40 W UV (Figure 8) fluorescent lamps. The test followed the guidelines established by ASTM G154 [15]. Three test specimens measuring 70 × 50 mm were used, where the equipment was set for cycles of 8 h of UV radiation and 8 h of condensation (rain). This cycle was repeated until 504 h were completed at a temperature of 70 °C.

The resistance to salt crystallization was evaluated according to the UNE-EN 12370 [16], using six specimens with dimensions of 40 × 40 mm. The samples were initially dried at 105 °C, immersed in a 14% Na_2_SO_4_ solution and subjected to 15 saturation and drying cycles. For the weathering test, 5 specimens with dimensions of 60 × 60 mm were used. The stones were exposed to weathering for 180 days.

**Figure 9:**
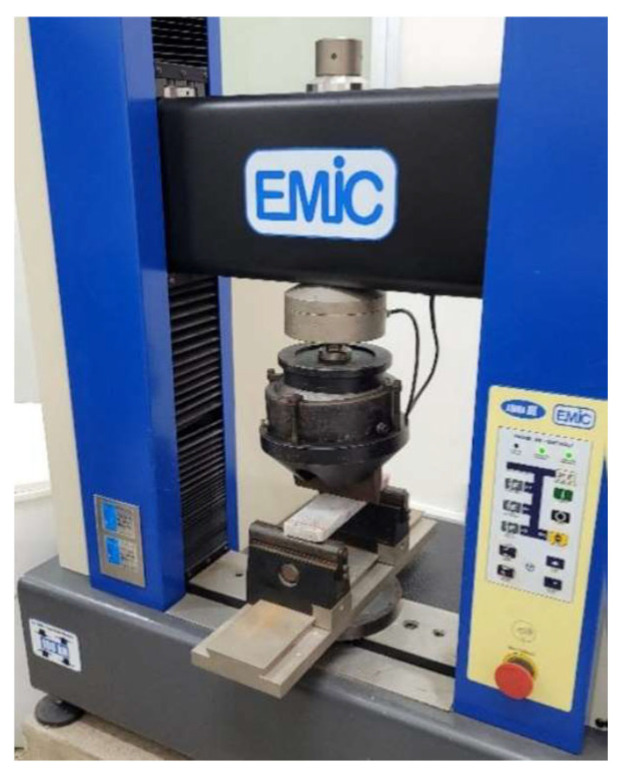
Universal testing machine (EMIC DL 10000).

To evaluate the effects of cycles on the determination of frost resistance and mechanical strength, a three-point bending test was performed with 5 specimens measuring 200 × 50 × 10 mm. The samples were subjected to 25 thermal cycles, each cycle consisting of a freezing stage at −15 °C for 16 h, followed by a thawing stage at room temperature (approximately 25 °C) for 8 h. After the cycles, the specimens were tested in a universal testing machine (manufactured EMIC DL 10000, Pinhais, PR, Brazil) (Figure 9), according to the requirements established by NBR 15845-4 [17].

## 3. Results and Discussion

### 3.1. Determination of the Particle Size Composition of the Highest Packing

Table 3 shows the dry apparent density results for each mixture of the simplex-centroid planning for the Preto São Gabriel granite waste and the Dumont Quartzite.

To verify if there is a significant difference between the means of each mixture, the results presented in Table 3 were subjected to an analysis of variance (ANOVA). The ANOVA was performed for the wastes of Dumont Quartzite and Preto São Gabriel granite.

Through the analysis of variance, the calculated F values of 330.59 and 105.06 were found for the wastes of the Dumont Quartzite and Preto São Gabriel granite stones, respectively. Comparing the calculated F values with the tabulated F value (2.39), at a significance level of 5%, it is verified that all of the calculated F values were higher than the tabulated F value, that is, it is concluded that there is a significant difference between the means of the dry apparent densities of the analyzed mixtures.

To verify which means show a significant difference between each other, the Tukey test was performed. The results are presented in Table 4.

Table 3 shows that mixtures 8 and 7 had the highest apparent dry densities for the Preto São Gabriel granite waste. For the Dumont Quartzite waste, mixtures 9 and 8 had the highest apparent dry densities.

As shown in Table 3, mixture 8 showed the highest dry apparent density for the Preto São Gabriel waste, while mixture 9 was the one that obtained the highest value for the Dumont Quartzite waste. Thus, mixture 8 was chosen for the production of artificial agglomerated stone using the Preto São Gabriel waste, and mixture 9 for the Dumont Quartzite waste.

### 3.2. Determination of Stain Resistance

**Figure 10:**
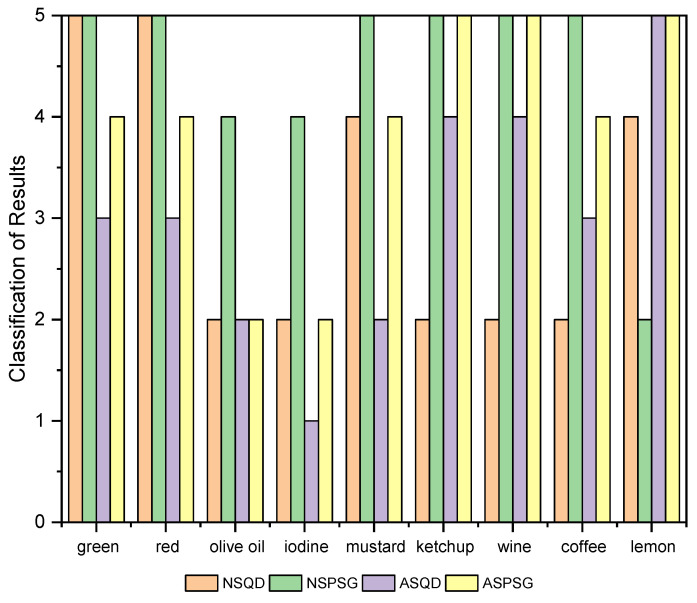
NSQD, NSPSG, ASQD and ASPSG stain resistance classification.

The purpose of the test is to simulate, under controlled laboratory conditions, the interaction between the stones and staining agents commonly used in cooking, cleaning and similar practices. This procedure is recommended to support the selection and appropriate use of stones in applications such as sinks, floors, countertops and tabletops. Figure 10 shows the results of determining the staining resistance of natural stones (NSQD and NSPSG) and artificial agglomerated stones (ASQD and ASPSG).

According to NBR 10545-14 [12], stain resistance is classified on a scale of one to five after exposure of stones to staining agents and successive attempts at removal. On this scale, the number five indicates that stains are easily removed (high stain resistance), and the number one indicates that stains are not removed after the stain removal steps.

At five, hot water is used to remove stains; at four, a neutral detergent is used; at three, an abrasive paste is used; at two, removal is done with a solution containing one of three agents: potassium hydroxide 200 g/L, hydrochloric acid or acetone; and finally, at one, if the stain persists after the previous steps, it indicates that it is impossible to remove the stains. The NSQD stone showed high stain resistance (scale 5) to green (Cr_2_O_3_) and red (Fe_2_O_3_) agents, which were easily removed with hot water. The mustard and lemon stains were removed with neutral detergent (scale 4). The olive oil, iodine, ketchup, wine and coffee stains were removed with potassium hydroxide (scale 2). No sample remained stained after the test.

The NSPSG stone showed high stain resistance (scale 5) to green (Cr_2_O_3_) and red (Fe_2_O_3_) agents, mustard, ketchup, wine and coffee, which were easily removed with hot water. The olive oil and iodine stains were removed with neutral detergent (scale 4). The lemon stain was removed with potassium hydroxide (scale 2). No sample remained stained after the test.

ASQD showed high stain resistance (scale 5) for lemon, which was removed with only hot water. The ketchup and wine stains were removed with neutral detergent (scale 4). The green (Cr_2_O_3_), red (Fe_2_O_3_) and coffee stains were removed using abrasive paste (scale 3). The olive oil and mustard stains were removed with a 200 g/L potassium hydroxide solution (scale 2). The iodine stain was the only one that remained after all of the cleaning steps.

ASPSG showed high stain resistance (scale 5) for ketchup, wine and lemon, which were removed with only hot water. The green (Cr_2_O_3_), red (Fe_2_O_3_), mustard and coffee stains were removed with neutral detergent (scale 4). The olive oil and iodine stains were removed with a 200 g/L potassium hydroxide solution (scale 2). No stains remained on the stone after cleaning.

When analyzing the results shown in Figure 2, the natural stones (NSQD and NSPSG) showed good resistance to staining. This fact can be attributed to the type of polished finish of these stones, as these stones have a smooth and high-gloss surface, thus contributing to the ease of stain removal.

The artificial agglomerated stone ASPSG showed good resistance to staining and, although it does not have the same type of polished finish as the natural stones, it has low porosity and water absorption. The stain caused by iodine on the ASQD stone was the only one that was not removed, and it was observed during the attempt to remove the stain that the iodine penetrated the surface pores of the material, thus making it difficult to remove. Ref. [18] states that the pores present on the surface of the stones are mainly responsible for the greater difficulty in removing stains.

Some studies have evaluated similar materials: Ref. [8] evaluated the stain resistance of their artificial stones developed with glass packaging waste, quartz powder, and epoxy resin. The same test method and staining agents were used, but the stone with iodine solution remained stained after all of the steps attempting to remove the stains. Ref. [18] produced an artificial stone by incorporating glass lamination waste into an epoxy matrix with the same staining agents. The stones produced did not present stains that could not be removed. Ref. [7] also performed a staining test on stones produced with granite and mirror waste. The stone that was attacked with the red agent remained stained after attempts to remove the stains. In a study carried out by [19], the stain resistance between natural and industrially produced artificial stones was compared, in addition to evaluating the loss of brightness after the test. The artificial stones showed approximately 15% greater effectiveness in resisting permanent stains compared to natural stones. Additionally, the natural stones showed 9.94% greater loss of gloss compared to the artificial stones.

### 3.3. Resistance to Chemical Attack

The chemical attack resistance test of natural and agglomerated stones visually assessed the chromatic modification, dissolution and mineral detachment, after the application of the reagents according to each attack time, according to the NBR 16596 [13] and Table 2.

**Figure 11:**
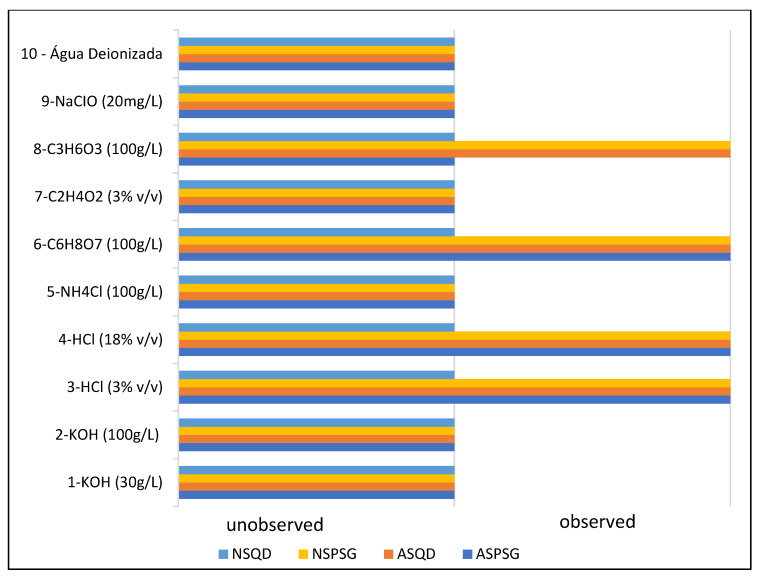
Chromatic modification—resistance to chemical attacks.

After visual analysis, there was no mineral dissolution or mineral detachment on the surfaces of the analyzed stones; however, a chromatic modification occurred in the natural and artificial agglomerated stones. Figure 11 shows the result of the chromatic modification for the analyzed stones.

According to the results shown in Figure 11, it can be seen that the natural and artificial agglomerated stones did not present chromatic alterations for the reagents: potassium hydroxide (KOH), ammonium chloride (NH_4_Cl), acetic acid (C_2_H_4_O_2_), sodium hypochlorite (NaClO) and deionized water.

It is possible to note that the most aggressive reagents were hydrochloric acid (HCl) at concentrations of 3% and 18%, followed by citric acid (C_6_H_8_O_7_) and lactic acid (C_3_H_6_O_3_). The results indicate the need to avoid contact of products containing hydrochloric acid, citric acid and lactic acid with stones. In addition to cleaning products, citric acid is found in citrus fruits and lactic acid in dairy products, and care should be taken to clean sinks and countertops after contact with these reagents. Figure 4 shows the chromatic modification of stones by hydrochloric acid (HCl) reagents at concentrations of 3% and 18%, citric acid (C_6_H_8_O_7_) and lactic acid (C_3_H_6_O_3_).

**Figure 12:**
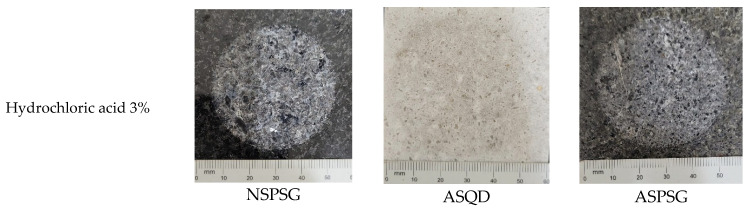

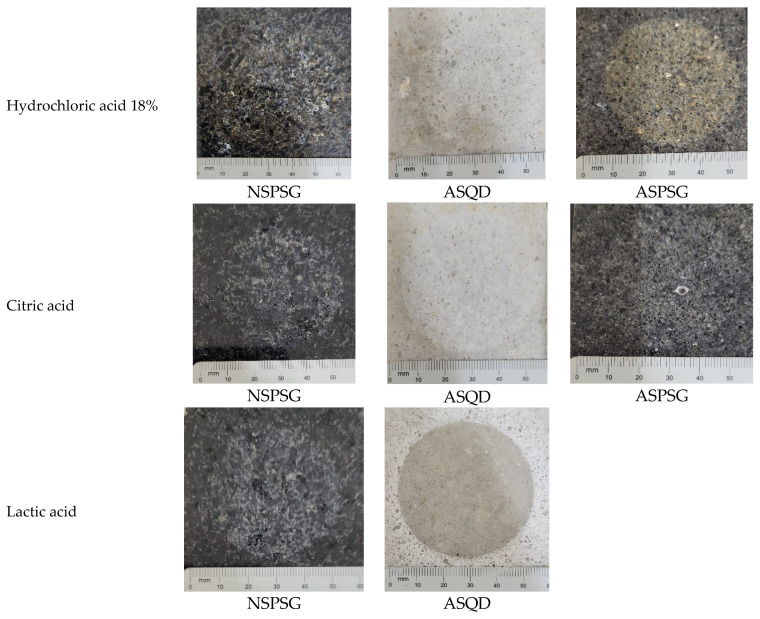
Chromatic modification of natural and artificial agglomerated stones.

As can be seen in the images in Figure 12, hydrochloric acid was the main culprit for the chromatic modification of natural and agglomerated artificial stones, followed by citric and lactic acid. In all stones visually analyzed, there was no dissolution or mineral detachment.

Ref. [7] produced an artificial stone by incorporating glass waste into an epoxy matrix with the same chemical agents. The stones produced showed a more intense chromatic modification for hydrochloric acid (HCl 3% and HCl 18%) and less intense for potassium hydroxide (KOH 30 g/L). This fact is justified by the high chemical resistance of epoxy resin to alkaline chemical agents [20].

Ref. [21] performed chemical attack tests using another methodology on artificial stones, using 80 wt% waste (quarry dust and chamotte from brick industries) and 20 wt% epoxy resin. The samples were in contact with chemical substances (NH_4_Cl, HCl, KOH, C_6_H_8_O_7_ and NaClO). In this study, it was possible to verify that HCl was the substance that resulted in the greatest weight loss in their stone samples, thus indicating that this agent was the most aggressive.

Ref. [8] produced artificial stones with glass waste and quartz powder in an epoxy matrix using NH_4_Cl, HCl, KOH and C_6_H_8_O_7_. In this study, they found that the samples exposed to alkaline substances showed less weight loss, and that the reagent that caused the greatest mass loss was HCl.

Ref. [22] produced artificial stones with 15 wt% castor oil polyurethane resin and 85 wt% granite with the agents NH_4_Cl, HCl, KOH and C_6_H_8_O_7_. They found greater mass loss in the stones that were attacked by hydrochloric acid and potassium hydroxide, since hydrochloric acid is a strong acid with a high degree of ionization. The authors do not recommend cleaning products that contain these acids, and only a damp cloth and diluted neutral soap are ideal for cleaning the stones. The results obtained in this study prove that hydrochloric acid was the most aggressive agent for stones, corroborating the results found in the literature. This type of chemical agent should be avoided in the composition of cleaning products for use on stones. Instead, it is recommended to use only a damp cloth with diluted neutral soap to maintain cleaning.

### 3.4. Wet and Dry Alterability

**Figure 13:**
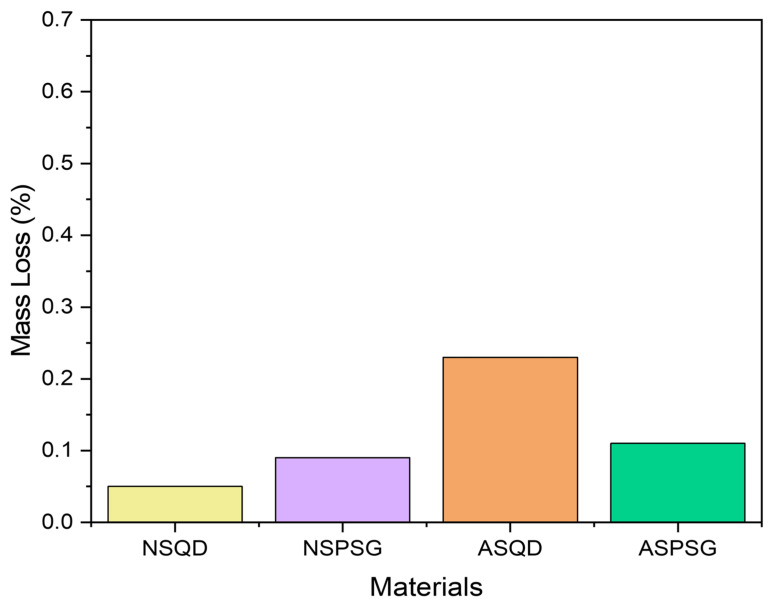
Mass loss after wetting and drying cycles.

This test is extremely important to assess the strength and alterability of stones when used in environments such as countertops, kitchens and bathrooms, where they are subject to humidity conditions and exposure to cleaning products [23]. Figure 13 shows the mass loss of natural and artificial agglomerated stones after accelerated degradation due to wetting and drying cycles.

It was observed that the artificial agglomerated stones had a greater loss of mass than the natural stones. The friction of the abrasive steel brush (hardness 5.5) on the artificial agglomerated stones during the test caused greater wear, since the minerals present in the natural stones have a greater hardness than the resin used to make the artificial agglomerated stones. Minerals such as quartz and feldspar, present in natural stones, have a hardness on the Mohs scale ranging from six to seven.

In addition, the porosity of the artificial agglomerated stones tends to increase with the brushing time, causing the minerals to become detached from the surface matrix of the stone, influencing the loss of mass [24].

The low porosity of the produced stones is directly associated with a denser and more homogeneous microstructure. This behavior suggests an efficient particle size distribution, favoring better packing and reducing void formation.

From a microstructural point of view, even without the direct presentation of images, it can be inferred that the matrix has good cohesion between the constituents, with a reduced presence of interconnected pores. The low porosity observed indicates that the remaining voids are predominantly isolated, which contributes to the improvement of the mechanical properties and alterability of the material.

Therefore, the combination of low porosity, compact microstructure, and well-adhered interface contributes to a material with superior performance, highlighting the effectiveness of the processing and formulation adopted.

An important point to consider is that the chemical bond of the resin with the stone aggregates for the formation of the artificial agglomerated stones may be weaker than the intrinsic chemical bonds between the minerals of the natural stones, leading to faster wear of the resin. This combination of factors can increase resin wear compared to minerals found in natural stones during this type of test.

Ref. [7] evaluated the mass loss in their stones produced with 85 wt% granite and glass waste in 15 wt% epoxy matrix and found a mass loss of 0.10 ± 0.05% for the Ocre Itabira granite and 0.12 ± 0.03% for the artificial stone. Ref. [23] produced artificial stones with 85 wt% quartzite and gravel (granite) waste in 15 wt% epoxy matrix and found a mass loss of 0.30%.

The results found for the natural and agglomerated artificial stones in this research are within the range of studies carried out by [7,23] (variation of 0.10% to 0.30% mass loss), indicating their suitability for application in areas prone to wetting and drying.

To verify if there was a significant difference in mass loss between the stones, an analysis of variance (ANOVA) was performed at a significance level of 5%. The ANOVA results are presented in Table 5.

The analysis of variance (ANOVA) revealed a highly significant effect of stone type and treatment on the mass loss of the samples (F3, 16 = 572.76; *p* < 0.0001). The magnitude of the F-value, presented in Table 5, allowed the rejection of the null hypothesis of equality between the means, confirming that the wetting and drying processes heterogeneously impacted the physical integrity of the stones. Therefore, to verify which ones presented a significant difference, a Tukey test was also performed at a significance level of 5%. The results are presented in Table 6.

Tukey’s test (α = 0.05) indicated that all treatments differed statistically from each other. When subjected to drying and wetting cycles, the ASQD stones showed the greatest average mass loss (0.234), followed by ASPSG (0.112) and NSPSG (0.094). The NSQD stone demonstrated the greatest stability, showing the lowest observed average mass loss (0.054). The low variation between repetitions (mean square of residual of 0.0000525) reinforces the consistency of the results obtained in the experiment.

### 3.5. Resistance to UV Radiation and Water Condensation by Accelerated Test

**Figure 14:**
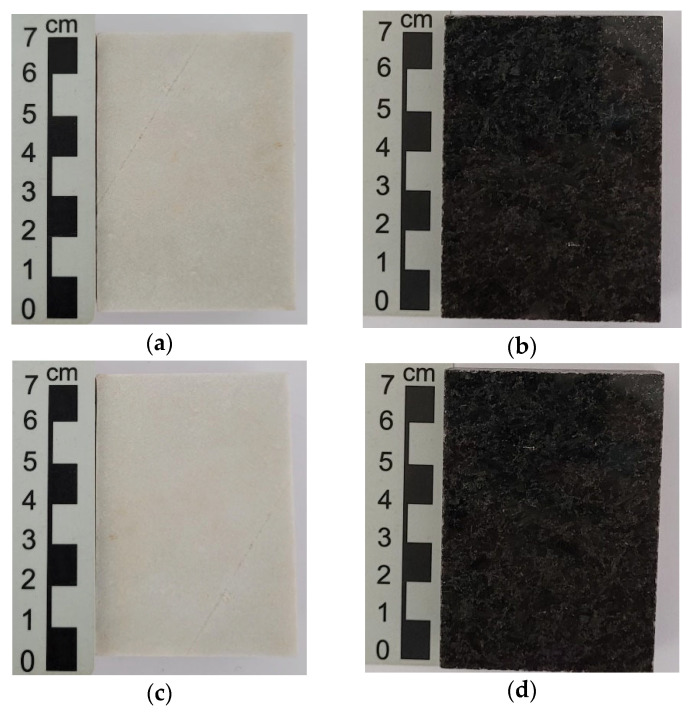
Natural stones before UV radiation: NSQD (**a**) and NSPSG (**b**). Natural stones after UV radiation: NSQD (**c**) and NSPSG (**d**).

Figure 14 shows the images before and after the UV radiation cycles for the natural stones (NSQD and NSPSG). As can be seen in Figure 14c,d, the natural stones studied are essentially of quartz-feldspar composition, and did not show any color changes due to UV radiation during the exposure time carried out in the study. The age and formation process of these stones, which occurred under extreme pressure and temperature conditions, contribute to their resistance to UV radiation and other adverse environmental factors [25,26].

**Figure 15:**
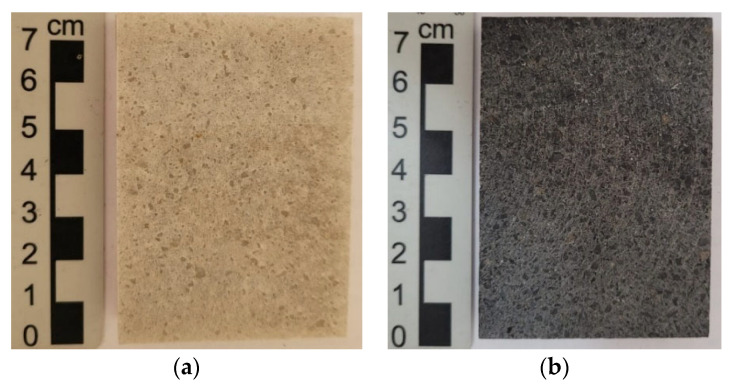

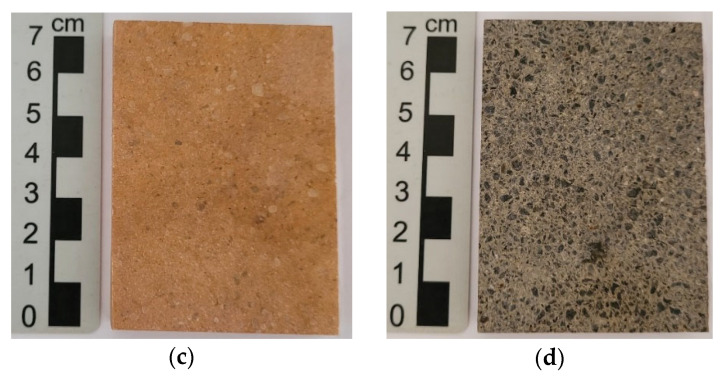
Artificial agglomerated stones before UV radiation: ASQD (**a**) and ASPSG (**b**). Artificial agglomerated stones after UV radiation: ASQD (**c**) and ASPSG (**d**).

Figure 15a–d show the images before and after the radiation cycles for the artificial agglomerated stones (ASQD and ASPSG). Figure 15c,d show a chromatic change on the surface of the samples of the artificial agglomerated stones after UV radiation, but no cracks or mineral detachment were visually observed. These results show that UV radiation affected the color of the epoxy resin on the surface of the sample. Prolonged exposure to UV radiation can compromise the surface integrity of the artificial stones, making them more susceptible to degradation.

Ref. [27] also analyzed their artificial stones produced with hazardous stone and sediment waste and found the presence of many microcracks on the surface of the samples after exposure to UV radiation. Although these cracks increased water absorption and caused color variation in the stones, they were not significant enough to reduce the strength of the artificial stone. Microstructural analysis revealed that UV radiation had a superficial effect on the samples.

### 3.6. Resistance to Salt Crystallization

To understand the properties of materials used in civil construction, assessing their alterability is extremely important due to their environmental exposure conditions. However, there are few studies on this topic. Figure 8 shows the mass loss of natural and agglomerated stones after 15 salt crystallization cycles. In each cycle, the samples were submerged in the anhydrous sodium sulfate solution and taken to the oven for drying, according to the specified durations.

**Figure 16:**
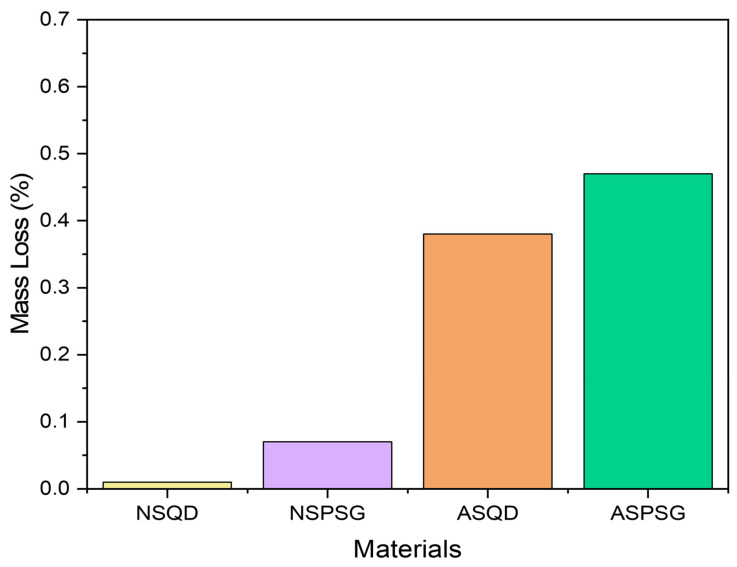
Mass loss after salt crystallization cycles.

Analyzing the data in Figure 16, it is possible to observe that the NSQD and NSPSG stones practically did not lose mass after the salt crystallization cycles. The NSPSG stone lost more mass because the mineralogical composition of this iron-magnesian stone includes minerals such as clinopyroxene and orthopyroxene. These minerals are reactive and less stable, and in the presence of saline solutions they can undergo chemical changes, even in low-porosity stones [28].

The artificial agglomerated stones (ASQD and ASPSG) showed a greater mass loss than the natural stones, due to the susceptibility of the epoxy resin to exposure to saline environments, especially in severe and prolonged conditions. Although the epoxy resin is chemically resistant, continuous exposure to anhydrous sodium sulfate can result in its degradation over time. Salts, present in all environments, crystallize by evaporation, expanding in the pores and causing internal tensions. The continuous cycle of liquefaction and solidification due to humidity causes microcracks and loss of cohesion between mineral grains. Degradation depends on the type of salt, the porous material and the environmental conditions.

A study carried out by [7] under the same conditions as this study obtained a mass loss of 1.36 ± 0.15% in the artificial stone produced with 82 wt% aggregate (quartz powder and steel waste) and 18 wt% epoxy resin from soybean oil. Ref. [8] produced artificial stones with glass waste and quartz powder in an epoxy matrix, subjected to 50 salt crystallization cycles, obtaining a mass loss of 0.87%.

To verify if there was a significant difference in the salt crystallization process and mass loss between the stones, an analysis of variance (ANOVA) was performed at a significance level of 5%. The ANOVA results are presented in Table 7.

The analysis of variance (ANOVA) indicated that the salt crystallization process promoted distinct changes in the treatments, with highly significant differences (*p* < 0.0001) between the observed mean mass loss. The high F value (819.56) corroborates the consistency of the data and the low influence of experimental error on the results.

Considering that the *p*-value was lower than the adopted significance level (α = 0.05), the null hypothesis was rejected, confirming that the treatment methods (NSQD, NSPSG, ASQD and ASPSG) impacted the resistance of the samples to salt attack unevenly. To identify which groups presented specific differences, Tukey’s test was applied (Table 8).

Tukey’s test (α = 0.05) indicated that all treatments evaluated differed statistically from each other, with the differences between the means exceeding the minimum significant difference (MSD) of 0.0310. The ASPSG treatment showed the greatest average mass loss (0.4671), followed by the ASQD treatment (0.3773) and the NSPSG treatment (0.0718). The NSQD treatment demonstrated the greatest stability against salt crystallization.

### 3.7. Weathering Resistance

**Figure 17:**
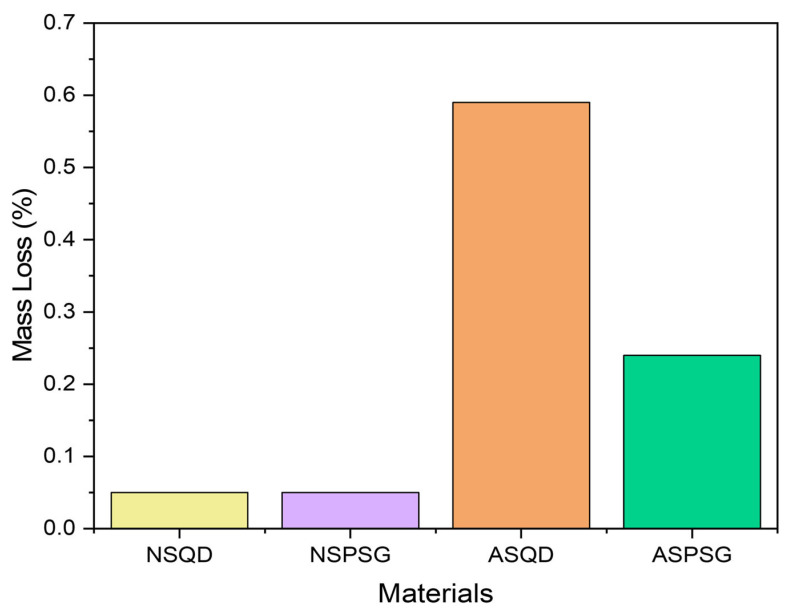
Mass loss after weathering.

Figure 17 shows the mass loss of natural artificial agglomerated stones exposed for 180 days to adverse weather conditions, such as prolonged exposure to the sun, rain, wind and other atmospheric agents that can accelerate their wear.

The tests were conducted during the summer and autumn seasons in the Cachoeiro de Itapemirim region, in the state of Espírito Santo. During the summer, typical environmental conditions include average temperatures between 26 °C and 35 °C and relative humidity between 75% and 90%. In the autumn, these conditions vary between 22 °C and 26 °C for temperature and between 65% and 80% for relative air humidity.

**Figure 18:**
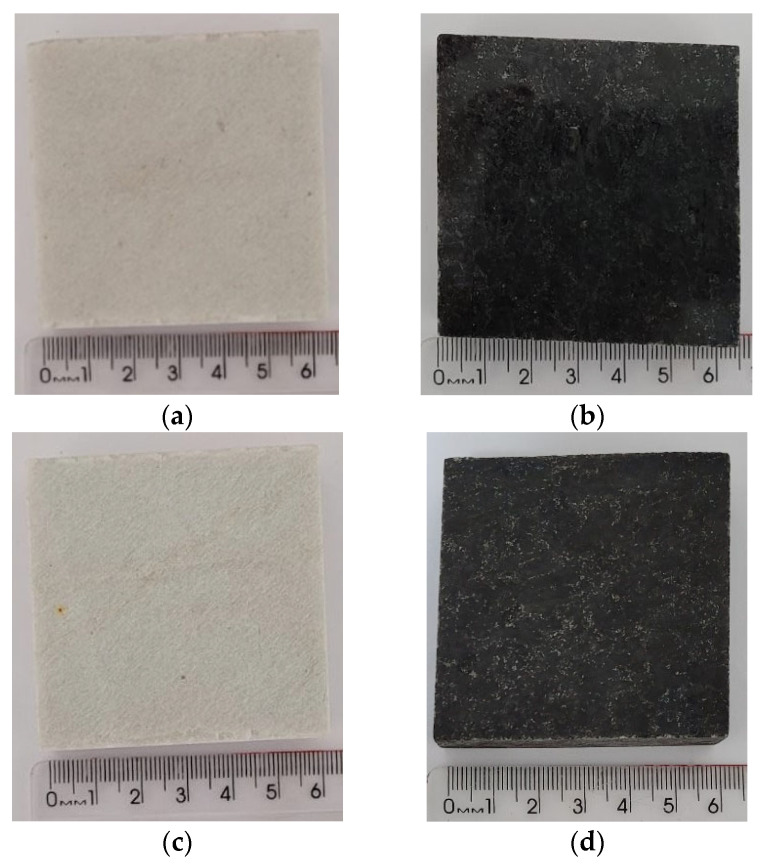
Natural stones before weathering: NSQD (**a**) and NSPSG (**b**). Natural stones after weathering: NSQD (**c**) and NSPSG (**d**).

The natural stones (NSQD and NSPSG) showed low mass loss and did not present chromatic or brightness changes in the samples analyzed as shown in Figure 18c,d after weathering, preserving their aesthetic characteristics even after exposure to the elements.

The time required for a stone to weather depends on several factors, especially the susceptibility of its constituent minerals and the climate. In less aggressive weathering conditions, a longer period of exposure is required for a weathering profile to develop [29].

**Figure 19:**
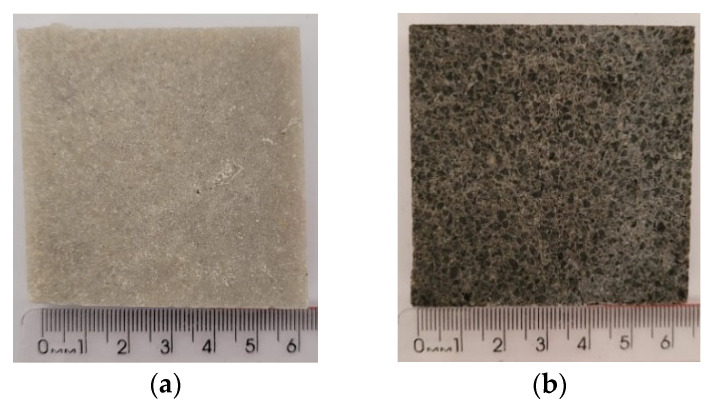

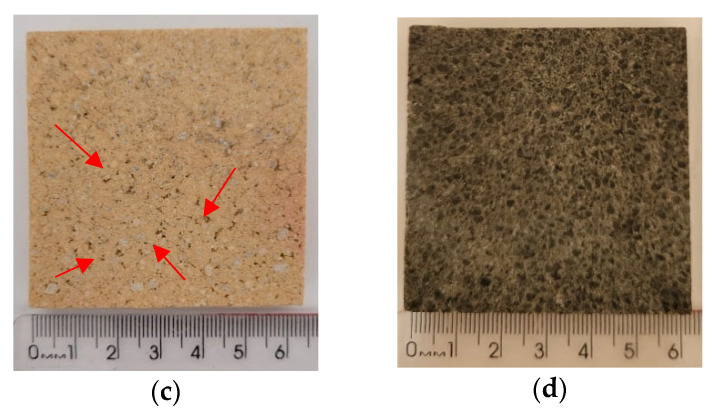
Artificial agglomerated stones before weathering: ASQD (**a**) and ASPSG (**b**). Artificial agglomerated stones after weathering: ASQD (**c**) and ASPSG (**d**).

On the other hand, the artificial agglomerated stones (ASQD and ASPSG) showed greater mass loss (Figure 17). In addition, chromatic changes were observed as shown in Figure 19c,d after exposure to different atmospheric conditions. These changes are attributed to the susceptibility of the epoxy resin to weathering, which can lead to surface degradation and aesthetic changes in the stones.

The presence of visible cracks and mineral detachment (indicated by the red arrows in Figure 19c) in ASQD (Figure 19c) highlights the reason why this stone presented a greater mass loss when compared to ASPSG (Figure 17). It is likely that factors such as acid rain, pollution and adverse weather conditions contributed to these effects. Although epoxy resin is effective in many applications and has good chemical resistance, its continuous exposure to adverse environmental factors can compromise its aesthetic and structural properties over time.

Applying specific sealants with UV protection additives can help extend the service life of the stone surface, maintaining its appearance and integrity, in addition to preventing yellowing, loss of shine and structural compromise. Implementing these measures can minimize the adverse effects of sun exposure, preserving the aesthetic and functional properties of artificial stones, as well as natural stones that require the application of resin.

From a chemical standpoint, this alteration can be attributed to photodegradation processes of the epoxy resin, in which UV radiation induces the breakage of polymer chains and the formation of free radicals. These radicals react with atmospheric oxygen, triggering photo-oxidation reactions, which lead to the formation of chromophore groups, such as carbonyls, responsible for the yellowing and color change observed. Furthermore, these processes can result in a slight modification of the surface structure of the matrix, without necessarily generating visible cracks on a macroscopic scale.

To verify if there was a significant difference in the weathering process and mass loss between the stones, an analysis of variance (ANOVA) was performed at a significance level of 5%. The ANOVA results are presented in Table 9. Table 10 presents the analyses from Tukey’s test.

The analysis of variance for the natural cycling test indicated highly significant differences between the stones analyzed (*p* < 0.0001). Tukey’s test revealed that the samples of Natural Dumont Quartzite (NSQD) and Natural Preto São Gabriel (NSPSG) presented the lowest mass loss indices, being statistically similar to each other (Group c).

In contrast, the artificial stones showed greater alterability, with ASQD presenting the highest average degradation index (0.5848), followed by ASPSG (0.2368). These results suggest that stones in their natural state have greater resistance to the thermal and moisture cycles of natural cycling, while the artificial versions demonstrate a greater susceptibility to physical weathering.

Quantitative analysis of natural weathering and UV radiation indicated that, although structurally stable, the stones undergo chromatic changes and greater mass loss (up to 0.5848 in ASQD) compared to natural stones, due to photodegradation of the epoxy resin. Natural weathering tends to intensify these mechanisms due to the combined action of radiation, humidity, and thermal variations, which favor processes such as hydrolysis and microcracking of the matrix, amplifying degradation when compared to natural stones, whose mineral phase is more stable to UV radiation.

### 3.8. Three-Point Flexural Strength Test After Freezing and Thawing

Table 11 shows the results obtained from the three-point flexural strength test and the loss of mechanical strength before and after the freezing and thawing cycles. To assess whether there was a change in the mechanical behavior of the stones, the test was performed under the same conditions used in the flexural strength at room temperature, with the aim of comparing the results.

**Figure 20:**
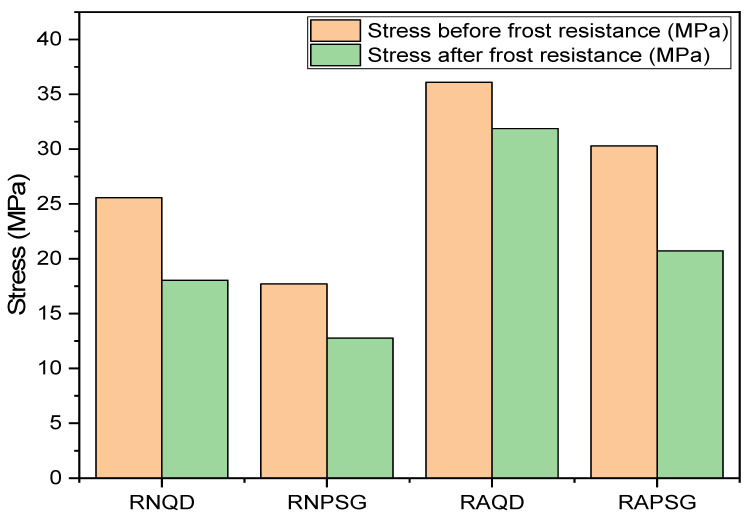
Flexural strength before and after freeze–thaw cycles.

Figure 20 shows a comparison between the results before and after the freeze–thaw cycles. As can be seen in Table 11 and Figure 20, both natural stones (NSQD and NSPSG) and artificial agglomerated stones (ASQD and ASPSG) showed a reduction in mechanical strength after the freezing and thawing cycles. Despite this reduction, all artificial agglomerated stones continue to meet the minimum requirement of NBR 15844 [30] and can be safely applied in civil construction. After the test, no visible changes were detected in the appearance of the stones, indicating that the mechanical changes occurred internally, without causing noticeable surface damage.

Artificial agglomerated stones (ASQD, ASPSG) maintained a mechanical strength above 20 MPa, being considered to have very high strength, according to the classification proposed by [31]. The freezing of water in stone fissures, which results in an increase in volume of approximately 9%, exerts pressure on the walls of the fissures. This pressure generates tensions that eventually fragment the stone and expand the fracture network.

The presence of open pores and microcracks in stone materials favors their deterioration over time, resulting in a decrease in mechanical strength. Water is identified as the main weathering agent, influencing the storage and circulation of fluids within these materials [32]. In freezing and thawing cycles, stones become susceptible to pressures generated by the expansion of water when freezing and contraction when thawing, reducing the mechanical strength of the stone [33].

In Ref. [21], freezing and thawing artificial stones, produced with 85 wt% granite waste with 15 wt% vegetable polyurethane resin from castor oil, resulted in a loss of mechanical strength of approximately 42% after testing under the same conditions as this study. The results of this research clearly indicate that the presence of water in the pores of the stone decreases its mechanical strength.

To assess the mechanical integrity of the stones, the flexural strength results were compared before and after exposure to freeze–thaw cycles. Table 12 presents the percentage variation in strength for each stone. The differences between the pre- and post-test states were validated via Student’s *t*-test, highlighting which treatments experienced statistically significant degradation in their mechanical properties.

The comparison between flexural strength before and after freeze–thaw cycles revealed distinct degradation behaviors among the stones. The ASQD stone demonstrated the greatest resilience to freeze/thaw cycles, showing a non-significant reduction of only 11.67%. In contrast, the ASPSG treatment exhibited the greatest vulnerability to thermal cycles, with a sharp drop of 31.60%, suggesting that the stresses generated by ice crystallization in the voids of the artificially treated stone severely compromised its internal cohesion. The natural stones (NSQD and NSPSG) also showed significant reductions.

The interpretation of these results is based on the principles of materials science. The observed water absorption may be associated with the presence of pores and the connectivity between them, since materials with a larger volume of voids tend to facilitate fluid penetration. This behavior is described for composite materials, in which open porosity and the network of microcracks exert a direct influence on fluid transport and material alterability [27].

The degradation observed after exposure to ultraviolet radiation can be attributed to photo-oxidative processes in the polymeric matrix, which promote the breakage of molecular chains and result in loss of structural integrity and aesthetic alterations. This behavior is widely reported for polymeric materials exposed to external environments [20].

The effects observed in the salt crystallization test may be related to the generation of internal stresses resulting from the growth of crystals in the pores of the material. This process can induce microcracking and material loss, being one of the main degradation mechanisms in porous materials [8].

Degradation associated with freeze–thaw cycles can be explained by the expansion of water inside the pores during freezing, generating internal stresses that can result in progressive cracking. This mechanism is particularly relevant in materials with higher water absorption and connected porosity [32,33].

## 4. Conclusions

Regarding the staining test, the ASPSG stone did not show any stains after the cleaning procedures and can be used in environments subject to the penetration of liquids, such as bathrooms and kitchen sinks. The ASQD stone remained stained only with iodine.

In the chemical resistance test, the stones demonstrated resistance to most of the reagents tested, except for hydrochloric and citric acids, which caused color changes and stains on the surface. Therefore, it is essential to adopt preventive measures when using products containing these reagents, to avoid damage to the integrity of the stones.

In the alterability tests for wetting and drying cycles and salt crystallization, the stones showed good results, with no significant changes in mass loss. However, exposure to UV radiation and weathering caused color changes, suggesting its use in indoor environments, since the degradation of the epoxy resin can compromise the aesthetics of the stone over time.

In the determination of frost resistance, a decrease in the mechanical strength of the stones was observed; however, they maintained a very high strength classification, with values above 20 MPa.

As for future perspectives, microstructural analysis is recommended for a better understanding of the degradation mechanisms. Additionally, studies involving the application of sealants and waterproofing agents are recommended as a strategy to reduce surface degradation and increase the alterability of these materials, contributing to their application in more severe conditions.

In terms of practical implications, the results obtained indicate that the developed artificial agglomerated stones exhibit adequate performance for applications in indoor environments, demonstrating good chemical and mechanical stability. However, susceptibility to UV radiation and weathering, observed through chromatic alterations, may restrict their use in outdoor exposure conditions.

This study has limitations associated with the absence of microstructural characterization, which restricts the detailed correlation between degradation mechanisms and the matrix–particle interface. Additionally, the interpretation of long-term alterability is limited by the lack of specific technical standards that define acceptable limits for mass loss in artificial rocks during salt crystallization tests and wetting and drying cycles.

## Figures and Tables

**Table 1:** Granulometric ranges of the waste.

Granulometry	Grain Size (mm)	Waste
Coarse	2.00–0.425	Large size piece of stone from the block sawing
Medium	0.425–0.075	Large size piece of stone from the block sawing
Fine	<0.075	FIBRO

**Table 2:** Reagents, concentration and exposure times during chemical attack.

Code	Reagents	Concentrations	Contact Time (h)
1	KOH (Potassium Hydroxide)	30 g/L	96
2	KOH (Potassium Hydroxide)	100 g/L	96
3	HCl (Hydrochloric Acid)	3% *v*/*v*	96
4	HCl (Hydrochloric Acid)	18% *v*/*v*	96
5	NH_4_Cl (Ammonium Chloride)	100 g/L	24
6	C_6_H_8_O_7_ (Citric Acid)	100 g/L	24
7	C_2_H_4_CO_2_ (Acetic Acid)	3% *v*/*v*	24
8	C_3_H_6_O_3_ (Lactic Acid)	100 g/L	24
9	NaClO (Sodium Hypochlorite)	20 mg/L	24
10	Deionized Water	-	96

**Table 3:** Dry bulk density results for each simplex-centroid mixture design combination.

Mixture	Coarse (%)	Medium (%)	Fine (%)	Dumont Quartzite (g/cm^3^)	Preto São Gabriel Granite (g/cm^3^)
1	100	0	0	1.442 ± 0.022	1.590 ± 0.021
2	0	100	0	1.581 ± 0.006	1.602 ± 0.023
3	0	0	100	1.110 ± 0.015	1.452 ± 0.019
4	50	50	0	1.714 ± 0.036	1.790 ± 0.052
5	50	0	50	1.508 ± 0.030	1.855 ± 0.029
6	0	50	50	1.457 ± 0.014	1.708 ± 0.029
7	33	33	33	1.740 ± 0.028	1.920 ± 0.027
8	67	17	16	1.761 ± 0.013	1.995 ± 0.031
9	16	67	17	1.819 ± 0.018	1.839 ± 0.012
10	17	16	67	1.304 ± 0.013	1.703 ± 0.024

**Table 4:** Tukey test for average dry apparent density (*p* < 0.05).

Dumont Quartzite		Preto São Gabriel Granite	
Mixture	Averages (g/cm^3^)	Mixture	Averages (g/cm^3^)
9	1.82 ^a^	8	1.99 ^a^
8	1.76 ^ab^	7	1.92 ^ab^
7	1.74 ^b^	5	1.85 ^bc^
4	1.71 ^b^	9	1.84 ^bc^
2	1.58 ^c^	4	1.79 ^c^
5	1.51 ^d^	6	1.71 ^d^
6	1.46 ^de^	10	1.70 ^d^
1	1.44 ^e^	2	1.60 ^e^
10	1.30 ^f^	1	1.59 ^e^
3	1.11 ^g^	3	1.45 ^f^

Means with different letters in the same column indicate significant differences (Tukey, *p* < 0.05).

**Table 5:** ANOVA: analysis of variance.

Source of Variation	GL	Value of Squares	Medium Square	F Calculated	*p*-Value
Stones Analyzed	3	0.0902	0.03007	572.26	<0.0001
Waste	16	0.00084	0.0000525	-	-
Total	19	0.09104	-	-	-

**Table 6:** Mean mass loss and results of Tukey’s multiple comparisons test (α = 0.05) for the different stone types after wetting and drying.

Treatment	Average	Groups (Tukey 5%)
ASQD	0.234	a
ASPSG	0.112	b
NSPSG	0.094	c
NSQD	0.054	d

**Table 7:** ANOVA: analysis of variance.

Source of Variation	GL	Value of Squares	Medium Square	F Calculated	*p*-Value
Stones Analyzed	3	0.905	0.3016	819.56	<0.0001
Waste	20	0.0073	0.000368	-	-
Total	23	0.9123	-	-	-

**Table 8:** Mean mass loss and results of Tukey’s multiple comparisons test (α = 0.05) for the different types of stones after salt crystallization.

Treatment	Average	Groups (Tukey 5%)
ASQD	0.4671	a
ASPSG	0.3773	b
NSPSG	0.0718	c
NSQD	0.011	d

**Table 9:** ANOVA: analysis of variance.

Source of Variation	GL	Value of Squares	Medium Square	F Calculated	*p*-Value
Stones Analyzed	3	0.75504	0.25168	453.47	<0.0001
Waste	12	0.00666	0.000555	-	-
Total	15	0.7617	-	-	-

**Table 10:** Mean mass loss and results of Tukey’s multiple comparisons test (α = 0.05) for different stone types after weathering.

Treatment	Average	Groups (Tukey 5%)
ASQD	0.5848	a
ASPSG	0.2368	b
NSPSG	0.0534	c
NSQD	0.0521	c

**Table 11:** Loss of flexural strength after freezing and thawing cycles.

Materials	Strength Before Cycles (MPa)	Strength After Cycles (MPa)	Loss of Resistance (%)
NSQD	25.57 ± 1.14	18.04 ± 2.80	29.45
NSPSG	17.70 ± 0.32	12.76 ± 0.64	27.91
ASQD	36.09 ± 1.92	31.88 ± 3.60	11.66
ASPSG	30.29 ± 2.15	20.72 ± 1.53	31.59

**Table 12:** Comparison of average flexural strength (MPa) and percentage drop index before and after freeze–thaw cycles.

Stone/Treatment	Initial Resistance (MPa)	Post-Freeze Resistance (MPa)	Fall (%)	Significance
NSQD	22.57	18.04	−20.07	*
NSPSG	17.7	12.76	−27.91	*
ASQD	36.09	31.88	−11.67	ns
ASPSG	30.29	20.72	−31.60	*

(ns) not significant; (*) significant at *p* < 0.05; by Student’s *t*-test.

## Data Availability

The original contributions presented in this study are included in the article. Further inquiries can be directed to the corresponding author.
